# Small vessel vasculitis and dry gangrene secondary to combined CTLA-4 and PD-1 blockade in malignant mesothelioma

**DOI:** 10.1186/s41927-021-00238-8

**Published:** 2022-02-08

**Authors:** Joanna Kefas, Catherine Harwood, Myles J. Lewis, Peter Szlosarek

**Affiliations:** 1 Department of Medicine, St Bartholomew’s Hospital, London, UK; 2 Department of Dermatology, Royal London Hospital, London, UK; 3 Department of Rheumatology, Barts and the London, London, UK; 4 Barts Cancer Institute, Queen Mary University of London, London, UK

**Keywords:** Mesothelioma, Immunotherapy, Immune related adverse events, Vasculitis, Case report

## Abstract

**Background:**

Malignant pleural mesothelioma (MPM) is a rare and aggressive tumour with an overall poor prognosis. In October 2020, first line treatment with the PD-1 antagonist nivolumab and the CTLA-4 antagonist ipilimumab for unresectable disease was FDA approved—the first approved treatment regime since 2004. Interim analyses from the phase 3 CHECKMATE-743 study shows improvements in overall survival. Skin-related toxicities are the most commonly reported any-grade treatment-related adverse event from combined nivolumab and ipilimumab therapy.

**Case presentation:**

Here we report a case of a 35-year-old white male who developed digital ischaemia secondary to small vessel vasculitis after receiving PD-1 and CTLA-4 blockade therapy for MPM. His progressive ischaemia became gangrenous, and he required multi-speciality input and treatment with prednisolone, prostacyclin, mycophenolate mofetil and hydroxychloroquine.

**Conclusions:**

Our case highlights the importance of early detection, intervention, and a multispecialty approach to managing such complications in order to minimise the associated morbidity and mortality.

## Background

Malignant pleural mesothelioma (MPM) is a rare and aggressive tumour of the thoracic pleura. Therapeutic options include surgery, radiotherapy, chemotherapy, and immunotherapy; however, the overall prognosis remains poor.

Since February 4th 2004, first line systemic therapy has been the combination of cisplatin and pemetrexed chemotherapy, with a median overall survival of 12 months. Recently, the first-line phase 3 CHECKMATE-743 study of cytotoxic T lymphocyte antigen 4 (CTLA-4) and programmed death receptor-1 (PD-1) blockade with ipilimumab and nivolumab respectively versus standard chemotherapy in non-resectable mesothelioma reported a median overall survival of 18 months for the former with FDA approval on October 2nd 2020 [1]. Prior to Checkmate-743, several earlier trials had tested CTLA4, PD-1 and PD-L1 antagonists for relapsed mesothelioma, with adoption of dual immune checkpoint blockade into treatment guidelines [2–5].

Immune checkpoint blockade yields disease control rates of 50–77% and most immunotherapy-related adverse events are generally reversible and manageable [1–5]. For instance, in the INITIATE phase II trial of ipilimumab and nivolumab, 34% of patients had a grade 3 or 4 adverse event (as per the Common Terminology Criteria for Adverse Events), and only one patient discontinued treatment because of toxicities [3]. In contrast, in the MAPS2 randomised trial, 14% of nivolumab group, and 26% of the combined group had a grade 3 or 4 adverse event and three treatment-related deaths [2]. Combination therapy is associated with increased toxicity, attributed to the interaction of CTLA-4 and PD-1 blockade. Skin-related toxicities are frequently seen, but to our knowledge vasculitis has not been reported in mesothelioma-specific trials.

## Case presentation

**Fig. 1:**
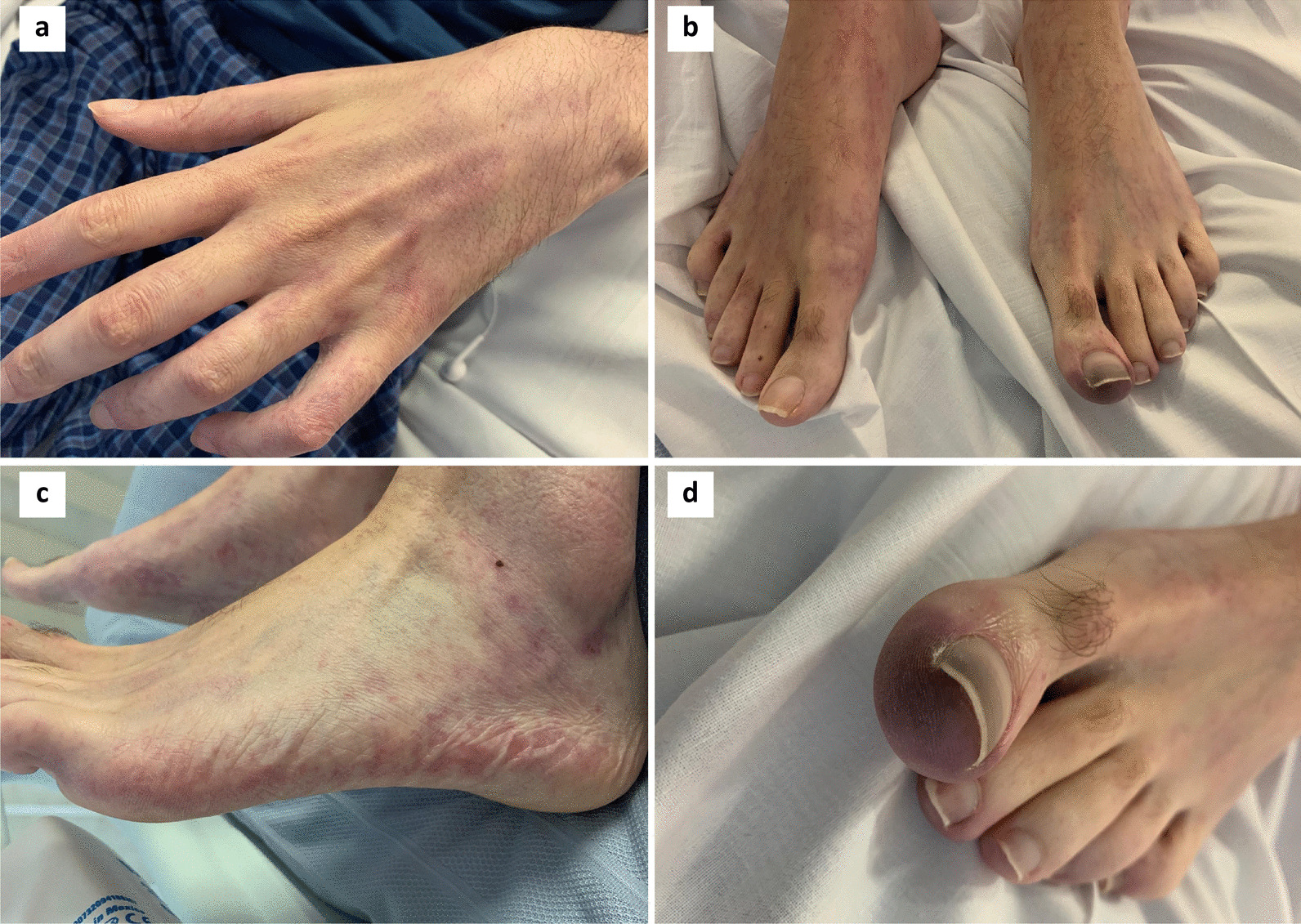
Photographs of described skin changes after 6 weeks of ipilimumab/pembrolizumab combination therapy. **a–c** Broken livedoid type skin changes symmetrically distributed in all four limbs. **d** A prominent area of purpura on the left hallux

We report on a 35-year-old white male who presented in April 2018 with a large, symptomatic pleural effusion secondary to a stage T2aN0M0 left-sided biphasic malignant pleural mesothelioma. He had no evidence of asbestos exposure nor the BAP1 inherited cancer syndrome. He declined enrolment into the MARS2 trial and proceeded in June 2018 to extra-pleural pneumonectomy (EPP) followed by palliative radiotherapy to his upper thoracic spine due to disease extending to the neural foramina. In September 2018, he developed a postoperative recurrence and was enrolled into the ATOMIC-meso trial, receiving a combination of pemetrexed, platinum and either placebo or the arginine-depleting agent ADI-PEG 20 until October 2019. He progressed and was then enrolled into the CONFIRM trial, a randomised study of nivolumab or placebo for relapsed mesothelioma. He was withdrawn after 3 months due to progressive disease clinically and radiologically. He developed a left renal vein thrombus and was started on low-molecular weight heparin. Due to worsening chest wall pain secondary to subcutaneous metastatic deposits, he was treated with 20 Gy in 5 fractions of radiotherapy in March 2020. Informed by his participation in the CONFIRM study, he then proceeded to combination immunotherapy with ipilimumab (1 mg/kg every 6 weeks) and pembrolizumab (2 mg/kg every 3 weeks) [6]. Within four weeks he noticed a subtle livedoid changes in his left hand, but no other associated localising symptoms. In May 2020, after 6 weeks the combination immune checkpoint blockade was discontinued due to significant progression of non-target disease including left subclavian artery compression by tumour. By this time the livedoid changes were extensively and symmetrically distributed in all four limbs but there was a particular prominent area of purpura on his left hallux (see Fig. 1). He was hospitalized to exclude acute limb ischaemia. He confirmed no history of autoimmune conditions, no prior dermatological history, and no associated symptoms of myalgia or arthralgia. The left subclavian artery was patent on CT imaging and the arterial supply to his lower limbs was normal by duplex doppler imaging with no evidence of embolic or thrombotic occlusion. A transthoracic echocardiogram showed no evidence of vegetation or significant valvular abnormalities. Serology was positive for speckled antinuclear antibody (ANA) with a titre of 1/640 and positive extractable nuclear antigens (Ro 60 positive and La weakly positive) and negative for rheumatoid factor, anticardiolipin antibodies, cryoglobulins, human immunodeficiency virus, anti-neutrophil cytoplasmic antibodies and double-stranded DNA; complement levels were normal. The patient declined a skin biopsy, and so a differential diagnosis of cholesterol embolism could not be excluded, although there was no evidence of hypocomplementaemia or eosinophilia during the development and progression of his skin symptoms. Overall, the clinical presentation and serology were consistent with immunotherapy-induced ANA positive small vessel vasculitis with digital ischaemia. He received a five-day course of intravenous methylprednisolone (1 mg/kg/day initially) followed by a tapering regimen of oral corticosteroids starting at 75 mg o.d. with good improvement, although it was not possible to reverse the dry gangrene now evident in the left hallux.

**Fig. 2:**
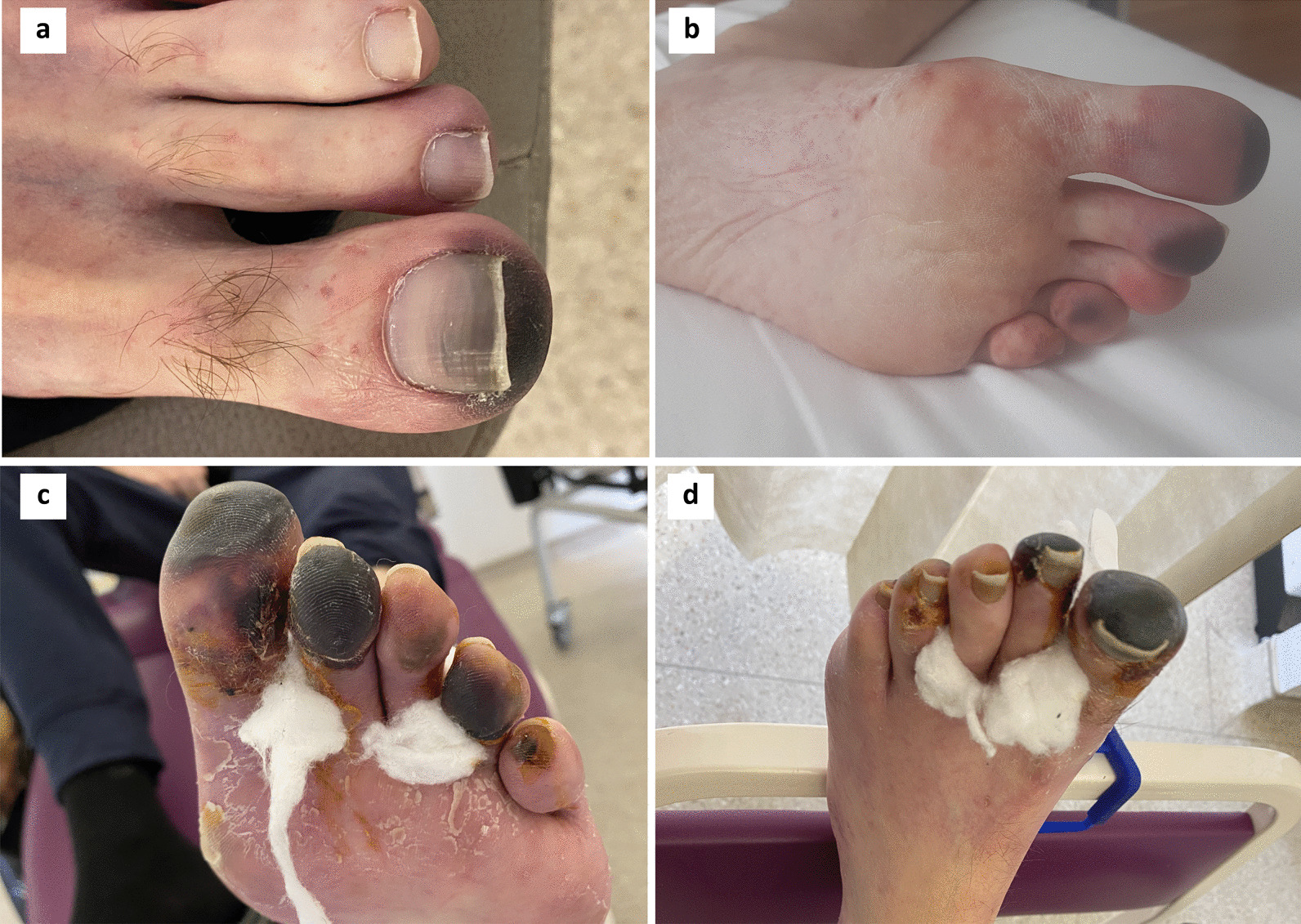
Follow up photographs of described skin changes over time. **a** Skin changes 5 weeks after first course of pulsed methyprednisolone and maintained on prednisolone 60 mg od. **b** 3 weeks after (**a**) and as steroid wean attempted, purpuric changes progressed to distal portions of other toes. Prednisolone re-escalated to 60 mg and slower weaning regime commenced. **c**, **d** 6 weeks after (**b**) slowly progressing necrosis and dry gangrene prompting second course of pulsed methylprednisolone and prostacyclin

To manage his mesothelioma progression, he started fourth-line treatment with gemcitabine and carboplatin chemotherapy resulting in weight gain, reduction in pain and dyspnoea, and a confirmed radiological response. Over the next two months his quality of life improved significantly. However, upon reducing his prednisolone to 30 mg o.d in August 2020 the dry gangrene in his left foot progressed to involve all 5 toes (see Fig. 2). Prednisolone was increased to 100 mg o.d. and he was admitted for a further 3-day course of pulsed intravenous methylprednisolone (1 mg/kg) and prostacyclin (epoprostenol 7 ng/kg/min) followed by prednisolone 100 mg/day, together with oral mycophenolate mofetil (750 mg b.d.) and hydroxychloroquine (200 mg b.d.). The vasculitis stabilised, but two weeks later he reported loss of taste, loss of smell, and increasing breathlessness and was hospitalised for COVID-19 pneumonia. Mycophenolate was withheld, he responded to treatment and was discharged but subsequently readmitted a week later with secondary bacterial pneumonia to which he succumbed in October 2020.

## Discussion and conclusions

With the expanding use of immune checkpoint inhibitors (ICIs), there is an increasing prevalence of diverse immune-related adverse events (IRAEs). These adverse events cover a broad spectrum of multi-organ involvement of varying severity and chronicity. The most commonly reported treatment-related adverse events are skin toxicities [1]. Although typically self-limiting, our case shows they can be unpredictable and cause significant morbidity and interruption to immunotherapy and conflicting requirements for immunosuppressive treatment strategies.

Vasculitis-type IRAEs are rare with less than 1% incidence, the most common of which are of large vessels (for example giant cell arteritis and aortitis) or central nervous system vasculitis [7]. A recent FDA Adverse Event Reporting System (FAERS) retrospective study of neurological IRAEs across multiple cancer types including small numbers of patients with mesothelioma, reported an increased risk with combined ICI therapy compared to monotherapy, but interestingly not in cases associated specifically with vasculitis [8].

However, there are increasing reports of acral ischaemia with the use of ICIs, with some resulting in digital amputation. To our knowledge, there have been three case reports of cutaneous small vessel vasculitis with ipilimumab use and four reports with pembrolizumab—but none in the context of mesothelioma [9]. In the cases reported, onset of symptoms ranges from 3 to 26 weeks and treatments used included high-dose steroids, aspirin, calcium channel blockers, prostaglandins and rituximab; outcomes were variable but in the majority of cases amputation was eventually required [10–12].

Given the mechanism of action of ICIs, and mounting evidence that they are associated with triggering increased autoimmunity-related reactions in susceptible individuals, larger studies are now needed to identify the aetiology of immunotherapy-related small vessel vasculitis and other ICI-linked autoimmune disease and determine optimal treatment strategies. There are theoretical concerns that immunosuppressive therapy may be associated with worse outcome due to malignant disease progression, but this remains to be proven. Guidelines for the best balance of treatment of both malignancy and ICI-linked autoimmune reaction still need to be determined to maintain survival benefits from ICIs and yet minimise IRAE. Prompt recognition and a multi-speciality collaborative approach is essential to minimise the associated morbidity and mortality.

## Data Availability

Not applicable.

## Abbreviations

MPM: Malignant pleural mesothelioma
CTLA-4: Cytotoxic T lymphocyte antigen 4
PD-1: Programmed death receptor-1
EPP: Extra-pleural pneumonectomy
ICIs: Immune checkpoint inhibitors
IRAEs: Immune-related adverse events
